# MS14, a Marine Herbal Medicine, an Immunosuppressive Drug in Experimental Autoimmune Encephalomyelitis

**DOI:** 10.5812/ircmj.16956

**Published:** 2014-07-05

**Authors:** Abbas Ebrahimi Kalan, Jafar Soleimani Rad, Laya Kafami, Daryoush Mohamadnezhad, Amir Afshin Khaki, Amaneh Mohammadi Roushandeh

**Affiliations:** 1 Anatomical Sciences Department, School of Medicine, Tabriz University of Medical Sciences, Tabriz, IR Iran; 2 Shefa Neuroscience Research Center, Tehran, IR Iran; 3 Pathobiology Department, School of Medicine, Alborz University of Medical Sciences, Karaj, IR Iran; 4 Anatomical Sciences Department, School of Medicine, Hamedan University of Medical Sciences, Hamedan, IR Iran

**Keywords:** Interleukin-6, EAE, Herbal Medicine

## Abstract

**Background:**

Cytokines are secreted signaling proteins which play essential roles in immune responses during experimental autoimmune encephalomyelitis (EAE), a demyelinating model that mimics many features of multiple sclerosis (MS). Interleukin 6 (IL-6) is a multifunctional cytokine produced by different cells, mediating inflammatory reactions and immune-mediated processes. Several studies have described immunosuppressive potentials of several herbal medicines. MS14 as an Iranian marine herbal medicine has anti-inflammatory and immunomodulatory activities.

**Objectives:**

The present study investigated the immunosuppressive potential of MS14 as an herbal drug as well as the IL-6 level in EAE model. We hope it will be a new approach for neurologic diseases and autoimmune originated diseases therapy.

**Patients and Methods:**

The present experimental study was a collaboration between Department of Anatomical Sciences of Tabriz University of Medical Sciences and Shefa Neuroscience Research Center of Tehran. We used 30 C57BL/6 mice. The animals were immunized with myelin oligodendrocyte glycoprotein (MOG) to induce EAE and treated with MS14-containing (30%) diets. Subjects were selected by simple random sampling and then they were randomly allocated to two groups. EAE symptoms were assessed using the standard 10–point EAE scoring system from the seventh to the 35th day after immunization. Afterwards, the spleen was removed and its cells were cultured with or without MOG 35-55; then, the IL-6 level was analyzed by ELISA. In addition, histopathological studies were carried out for demyelination lesion evaluation in the spinal cord.

**Results:**

MS14 significantly improved clinical symptoms of EAE compared with the control (P < 0.05). It also suppressed proliferative responses of T cells and decreased IL-6 expression (16.93 ± 2.7 vs. 21.4 ± 3.33) (P < 0.05).

**Conclusions:**

Our results strongly suggested that IL-6 as a potential molecule could have a role in neuroimmunology and neuroinflammation, which is in congruent with previous studies. Therefore, it can be a clear target in strategic therapies and support effective properties of phytotherapy in EAE and MS treatment.

## 1. Background

Multiple sclerosis (MS) is the most common chronic inflammatory debilitating disease of the central nervous system (CNS). MS is predominantly involved in immune-mediated demyelination as well as lesions that are pathologically caused by infiltrating macrophages and autoreactive T cells, particularly in the white matter. Current therapies for MS are not sufficient and this disease is affecting the young adults’ lives. Hence, scientists have been attempting to design therapeutic approaches targeting main immunopathological processes, which play crucial roles in MS pathophysiology. Increasing evidences show that cytokines and T-cells play important roles in mediating MS pathophysiology; thus, lots of efforts have focused on developing an efficient basis for future specific immune intervention strategies for MS and experimental autoimmune encephalomyelitis (EAE), a demyelinating animal model that mimics many clinical and pathological features of MS (1-8). Cytokines are secreted signaling proteins which play essential roles in propagating and regulating the immune responses during EAE. The inflammatory cytokine interleukin 6 (IL-6), originally identified as a T-cell-derived factor, induces antibody secretion and final maturation of B-cells (9). IL-6 is a multifunctional cytokine produced by a wide variety of cells, including macrophages, lymphocytes, fibroblasts, and various CNS cell types (10-13). In addition, it mediates inflammatory reactions and immune-mediated processes (13, 14). IL-6-deficient mice were completely resistant to EAE (1). MS treatment involves an immune suppressing process. The literature using EAE suggested that phytotherapies and antioxidants may even be curative for neurological diseases (15). Herbal remedies can prevent the progression of MS and improve its symptoms. MS14 as an herbal-marine compound has anti-inflammatory and immunomodulatory activities, including reduction of TNFα and IL-1β production (16, 17). However, it’s possible positive effects on MS have not been cleared yet.

## 2. Objectives

The purpose of this study was to examine the MS14 effects (18, 19) on clinical parameters including the IL-6 level in well-established animal models of MS (EAE), in experimental and control groups.

## 3. Materials and Methods

### 3.1. Materials

RPMI-1640 medium, penicillin/streptomycin, and fetal bovine serum (FBS) were purchased from Gibco (Invitrogen, Germany). Hooke kit for EAE induction (myelin oligodendrocyte glycoprotein_35-55_ (MOG_35-55_) (20) combined with complete Freund’s adjuvant (CFA) emulsion (21) and pertussis toxin (PTX):5X) and MOG_35-55_ for in vitro stimulation of cells were purchased from Hooke laboratories (EK-0115, Lawrence, MA, USA). Reagents required for histopathological analyses including paraffin, xylol, alcohol, phosphate buffer saline (PBS) and Luxol fast blue were obtained from Sigma (Sigma-Aldrich, Germany). IL-6 ELISA kit and cell proliferation ELISA BrdU kit were obtained from e-Bioscience and Roche, respectively. MS14, an Iranian herbal-marine compound classified as equivalent to food with no observable adverse effect level (NOAEL), was donated by deceased Dr. Ahmadi.

### 3.2. Animals

Thirty 8-10 weaks-old female C57BL/6 mice were purchased from Pasture Institute (Iran) and housed under standard humidity, 22-23°C temperature, and 12/12 (7 AM–7 PM) dark/light cycles in pathogen-free animal laboratory conditions. Each four mice were housed in one cage and maintained one week to acclimatize. The ethical committee of Tabriz University of Medical Sciences approved all the experiments with code 54\2588 on 2011/04/27. The randomized block allocation was used to allocate the subjects to groups. The random sequence was generated in blocks sized four, by the random allocations software.

### 3.3. Experimental Autoimmune Encephalomyelitis Induction

C57BL/6 mice were immunized with Hooke kits according to the manufacturer’s instruction. Briefly, after a mild anesthesia, 0.1 mL MOG_35-55_/CFA emulsion was subcutaneously injected to both flanks of each mouse (0.2 mL/animal); then, the animals received pertussis toxin on the same day and 24 hours later (0.1 mL/animal/day), intraperitoneally (20).

### 3.4. Treatment

The EAE-induced mice were randomly divided to control and MS14-treated groups. All animals were housed separately in each cage and MS14 was administrated to the treatment group orally. They were also fed with MS14-containing (30 %) diet from the day of immunization (19) to the end of the study.

### 3.5. Clinical Evaluation

Mice were weighed and examined daily for beginning of EAE symptoms, which was assessed using the standard 10–point EAE scoring system from the 7th to the 35th day of immunization by a single researcher blind to the treatment groups, as follows:

Zero, no obvious changes in motor functions;
0.5, partial tail paralysis;
1.0, complete tail paralysis;
1.5, complete tail paralysis and discrete hind limb weakness;
2.0, complete tail paralysis and strong hind limb weakness;
2.5, unilateral hind limb paralysis;
3, bilateral hind limb paralysis;
3.5, hind limb paralysis and forelimb weakness;
4.0, complete paralysis (tetraplegia);
5.0: moribund or dead (22, 23).

Three clinical parameters were analyzed to compare the course of EAE: A. severity of the disease with cumulative disease index (CDI), detected as sum of daily clinical scores during the disease; B. disease onset; C. peak disease score; i.e. the highest score reached by each animal during the course of disease (6).

### 3.6. Cell Culture

Twenty-one days after immunization, four mice were sacrificed from each group and their spleens were removed aseptically. A single cell suspension was prepared from each spleen according to the standard protocol. Briefly, the spleens were cut into small pieces and passed through 70 μm cell strainers. After washing, the cells were resuspended in 2 mL of media and cultured in round-bottom 96-well plates at 2 × 10^5^ cells per well density in triplicates. The culture medium contained RPMI-1640, 10% FBS, and 1% streptomycin/penicillin, with or without MOG_35-55_ (20 μg/mL). The plates were maintained in a 5% CO_2_ incubator at 37°C.

### 3.7. Cell Proliferation and Cytokine Production Analysis

After 48 hours, the plate was centrifuged at 1000 rpm for 10 minutes and half of the cells supernatant was removed and used for cytokine assessment. The cells were then resuspended and 10 μL of BrdU labeling solution was added to each well; then, the plates were maintained in the 5% CO_2_ incubator at 37°C for the next 72 hours. The cells were then transferred to 96-well plates and centrifuged at 1000 rpm for 10 minutes and the labeling medium was removed. Afterwards, BrdU proliferation assay was performed according to the manufacturer’s instruction, using cell proliferation ELISA BrdU kit (Roche, France). IL-6 was measured by ELISA (eBiosciences), according to the protocol recommended by the manufacturer.

### 3.8. Histopathological Analysis

After 35 days, the mice were deeply anesthetized with ketamine/xylazine (5/1). Following intracardiac fixative perfusion, the spinal cords were fixed with 4% paraformaldehyde for 72 hours. The tissues were treated with ethanol and xylene according to the routine protocol and five-µm sections were stained with Luxol fast blue to detect the demyelination area. Five randomly sections of spinal cord in each animal were photographed using microscope (Axioskop2; Carl Zeiss MicroImaging Inc.). Surface of the demyelinating area in each section was measured using INFINITY software version 4.6.0. For normalization, the mean surface was calculated in 1 mm^2^.

### 3.9. Statistical Analysis

Statistical significance of clinical signs was calculated by Mann-Whitney test between control and MS14-treated groups. For evaluation of cytokine levels and inflammation in spinal cord student’s t-test was used. P < 0.05 was considered statistically significant.

## 4. Results

### 4.1. MS14 Treatment, Recovery and Reduced Clinical Signs

**Figure 1.**
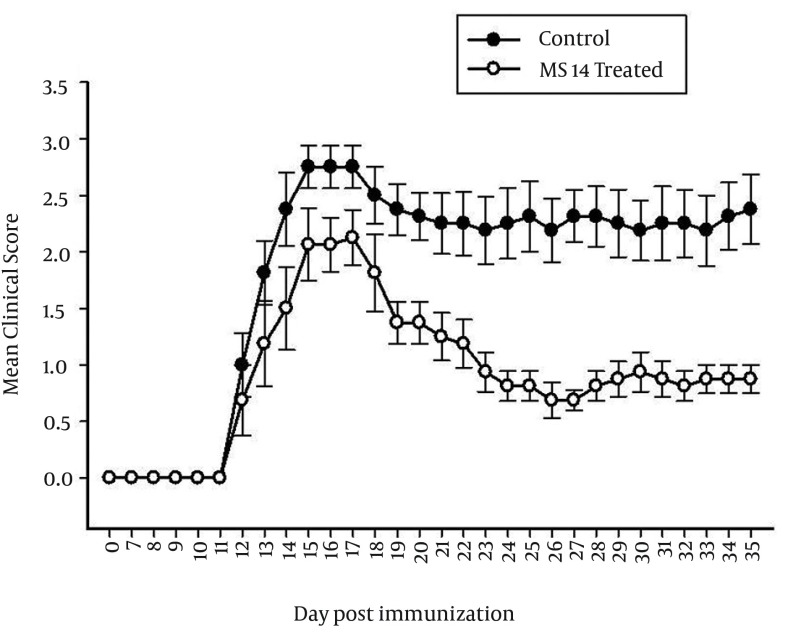
Clinical Course and Severity of Experimental Autoimmune Encephalomyelitis in Animals Received MS14 C57BL/6 mice were immunized with MOG_35-55_ peptide to induce EAE and were treated daily by MS14 orally. The mice were scored daily, as described in materials and methods.

To recapitulate MS, EAE was induced in C57BL/6 mice, using MOG. Daily clinical score of the disease was inhibited significantly by MS14 at the effector phase of the disease (P < 0.05, day 22-35 after immunization, Figure 1A). MS14-treated animals showed significantly lower EAE severity (36.43 ± 4.8) as measured by CDI, compared with the control animals (53.25 ± 4.88) (P < 0.05, Figure 1B). MS14 also stabilized the disease by reducing the severity of EAE (CDI 1.04 ± 0.13), which was significantly lower than the control group (CDI 1.51 ± 0.13) (P < 0.05,) (Figure 1).

The onset of clinical signs was approximately at 12 DPI (12 days after the boost with MOG peptide), reaching a peak stage at 21 DPI, and most animals remain ill (score 3-3.5) throughout the entire experimental period (35 DPI) (Figure 1). The highest clinical signs of EAE without any improvement for at least three uninterrupted days were defined as the acute phase of the disease. MS14 did not show any significant effects on clinical scores at the peak of the disease. Furthermore, it could not significantly postpone the disease unset. In the chronic phase (30^th^ to 35^th^ postimmunization), the clinical scoring rate was significantly lower in MS14-treated group compared with the control group (P < 0.05) (Figure 1).

### 4.2. MS14 Treatment Suppressing Neurodemyelination

**Figure 2.**
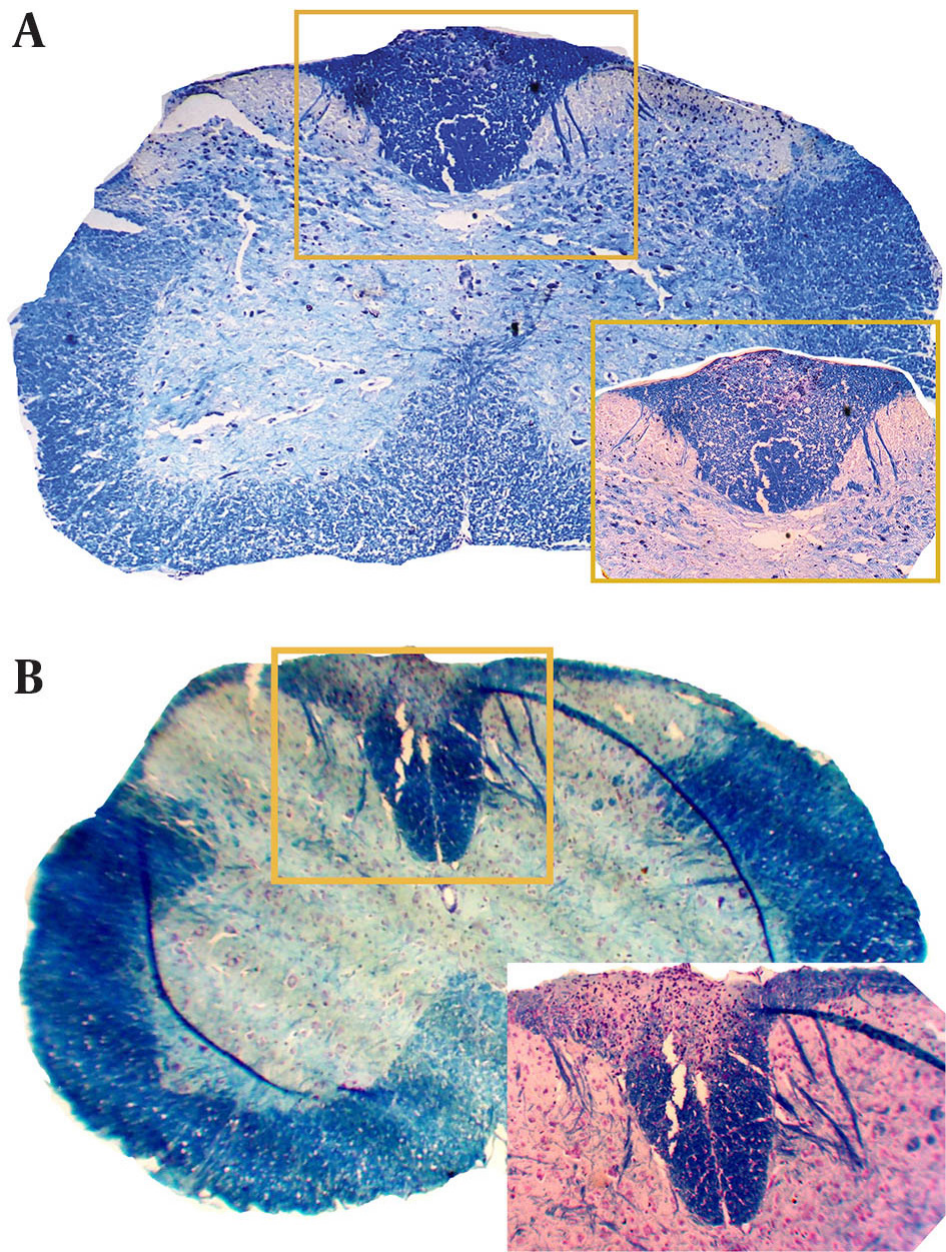
Demyelination in the Spinal Cord Evaluated by LFB Staining In the MS14-receiving group (B), the demyelinated area measurement in the spinal cord was attenuated signiﬁcantly in comparison with the control group (A) (P < 0.05).

**Figure 3.**
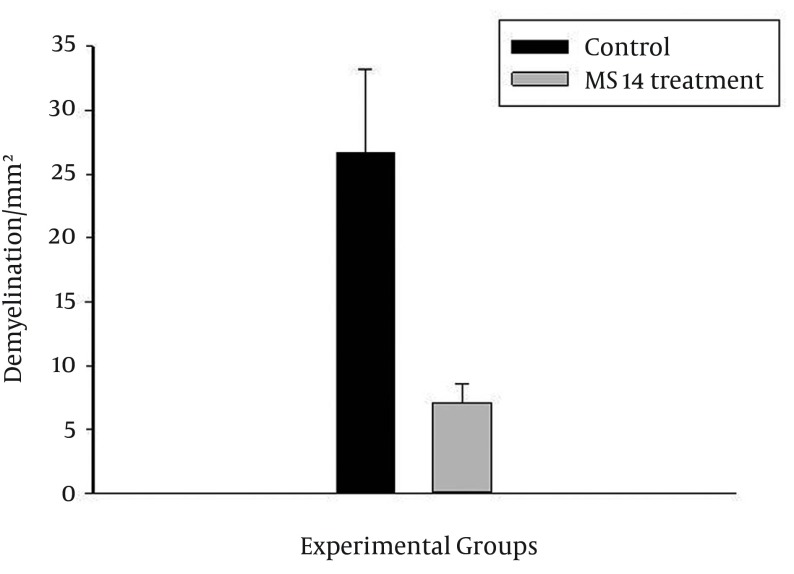
Demyelinating Area, Measured at Axial Section of Spinal Cord in MS14-Treated and Control Groups at 35th Day Post-immunization Values are shown as mean ± SEM (B, *pb 0.05, student's t-test).

The demyelinating area was assessed at this stage with Luxol fast blue staining. Demyelination, mostly within the white matter, analyzed the histopathology of spinal cord lesion burden (Figure 2A). Animals in the control group were sacrificed at 21 DPI (control young) and 35 DPI (control old) (Figure 2B). The measuring area of white matter indicated significant decrease of demyelinating area after MS14 treatment in EAE animals (P < 0.05) (Figure 3).

### 4.3. MS14 Treatment and T-Cell Function and Number

**Figure 4.**
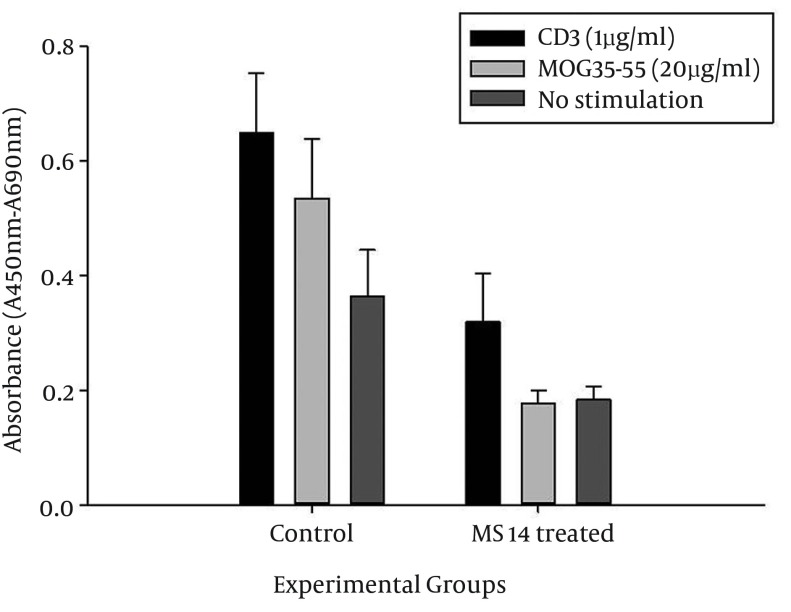
Effects of MS14 on T-cell Proliferation Proliferation of T-cells, pulsed with MOG_35-55_ and anti-CD_3_ antibody, was assessed at day 21 post-immunization by ELISA. Values are shown as mean ± SEM. Black, light gray and dark gray columns represent the amounts of BrdU incorporation into the DNA of anti-CD3, 20 μg/mL MOG_35–55_-stimulated, and unstimulated T-cells, respectively. Proliferative response of T-cells to anti-CD_3_ and MOG_35–55_ was suppressed by MS14 treatment. It also decreased the proliferation of splenocytes, compared with the unstimulated group of cells (*pb 0.05, student's t-test).

Effects of MS14 on T-cell proliferation have been investigated using in vitro MOG_35-55_ stimulation of splenocytes, isolated from MS14-treated and control animals. MS14 significantly suppressed the proliferative response of T-cells to MOG_35-55_, compared with the control group (P < 0.05) (Figure 4). In this study, we evaluated the non-antigen-specific response of T-cells, using anti-CD_3_ antibody stimulation in vitro. Considerably, MS14 covered up the proliferation in anti-CD3-pulsed T-cells significantly (P < 0.05) (Figure 4).

### 4.4. IL-6 Production as a Proinflammatory Cytokine, Suppressed by MS14 Treatment

**Figure 5.**
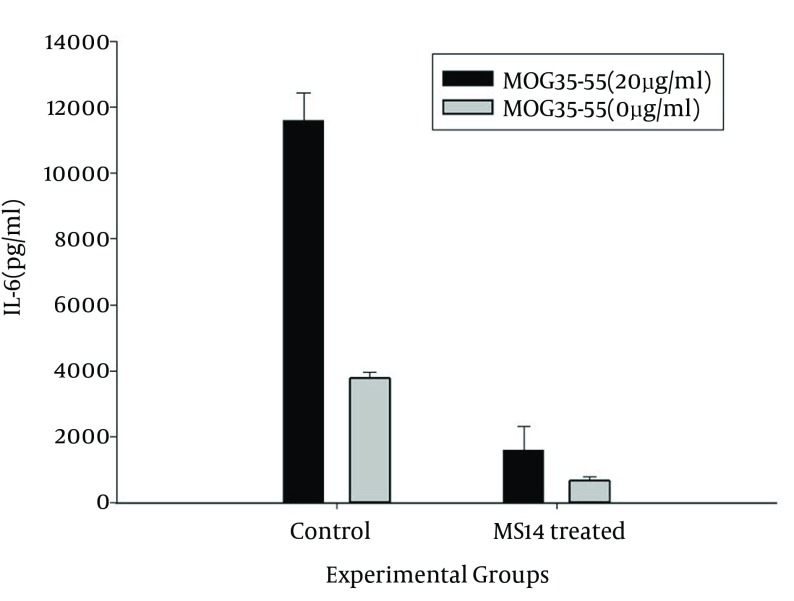
The Interleukin 6 Level, Produced by Splenocytes in the Groups Values are shown as mean ± SEM. Black and gray columns represent the cytokine levels for 20 μg/mL MOG_35–55_-stimulated and unstimulated T-cells, respectively. The MS14 group showed signiﬁcantly lower levels, compared with the control group (*pb 0.05, student's t-test).

Level of IL-6 as an inflammatory cytokine involved in the neuroinflammatory process was measured in supernatants of MOG_35-55_-stimulated splenocytes. ELISA showed a significant decrease in the level of IL-6 in MS14-treated group compared with the control group (P < 0.05) (Figure 5).

## 5. Discussion

In this study, we found that administration of MS14 attenuated clinical and histopathological features of EAE through immunosuppressive effects. MS14 inhibited proliferation of T-cells, stimulated by either MOG_35-55_ peptide or anti-CD3 antibody, which probably acts through immunomodulatory mechanisms. In addition, the clinical score was diminished in EAE mice treated with MS14. MS14 improved EAE symptoms more effectively at the chronic phase rather than the peak phase of the disease. Improvement of the clinical score was accompanied with reduction in demyelinating areas in the spinal cord. Pathogenesis of MS is still unknown and its full picture remains a mysterious disorder. Our results, similar to previous studies, strongly suggested that IL-6 as a potential molecule could have a role in neuroimmunology and neuroinflammation. We found that IL-6 that acts as a B-cell differentiation factor was reduced in EAE mice after MS14 treatment. IL-6 and another cytokines have important roles in inflammatory reactions. Based on previous studies, IL-6 not only is involved in immune responses, but also contributes to other important physiological functions in tissues including nervous system tissues. IL-6 takes part in neurogenesis of CNS cells and response of mature neurons and neuroglial cells in normal conditions as well as in the animal model of MS. IL-6-deficient mice have been resistant to EAE induction, which is involved by reactivated T-cells (24-26). IL-6 and IL-21 are complementary for production of Th17 cells as a subset of CD4^+^ T-cells, which plays a great role in EAE development. Microglia involves in production of cytokines such as IL-6 and TNF-α, which can cause more inflammatory cytokines production by CNS-reactive CD4^ (+)^ T-cells (5). Previous studies have demonstrated that glial cells can be the key effectors of EAE neurohistological changes (6, 27). Collectively, these data proved IL-6 as a clear target of strategic therapies such as herbal medications. In this research, MS14 as an herbal drug decreased the level of IL-6 and subsequently caused reduction of neuroinflammation and improved remyelination in the spinal cord. The recent treatments have targeted anti-inflammatory mechanisms to inhibit or slow down the disease progression. Identification of new anti-inflammatory agents to enhance axon myelination would present new therapeutic approaches to inhibit and possibly reverse the disease progression (27, 28). Our findings indicated that herbal drugs diminished the neurological signs of EAE, which was supported by prior studies (19, 29). MS14 as an anti-inflammatory remedy decreased T-cell proliferation and proinflammatory cytokines levels. A previous study revealed that MS14 modified innate and cellular immune responses as well as its anti-inflammatory effects during the disease (30). Phytotherapy could be curative with various pathways; i.e. it showed antifatigue properties in ginseng treated mice (31). In another study, orally treated mice with green tea showed altered proliferation of CD4^+^ T-cells in EAE. Regarding several studies that evaluated some herbal drug interrelates with immunologic conditions, modulation of a wide range of immunological parameters end to immunosuppression, matching our findings (26, 28).

Finally, our results demonstrated that MS14 could exert its mechanism via immunosuppressive activity. IL-6 level decreased during EAE, which could be an important strategy for treatment of EAE as a well-accepted animal model of MS; but more studies are required to elucidate the involved mechanisms.
